# Supplementary material for: Quantifying population contact patterns in the United States during the COVID-19 pandemic
Source: Nat Commun. 2021 Feb 9;12:893. doi: 10.1038/s41467-021-20990-2 (PMC7873309; doi:10.1038/s41467-021-20990-2)
Supplement: Supplementary file 1 — Supplementary Information [file 41467_2021_20990_MOESM1_ESM.pdf]

A Supplementary information

Supplementary table 1 shows detailed characteristics of the sample.

Supplementary table 1: Characteristics of the sample.

	Unweighted					Weighted				
	Overall, N = 9,743	Wave 0, N = 1,437	Wave 1, N = 2,627	Wave 2, N = 2,431	Wave 3, N = 3,248	Overall, N = 9,743	Wave 0, N = 1,437	Wave 1, N = 2,627	Wave 2, N = 2,431	Wave 3, N = 3,248
**Gender**										
**Female**	5,061 (52%)	756 (53%)	1,376 (52%)	1,256 (52%)	1,673 (52%)	5,001 (51%)	738 (51%)	1,348 (51%)	1,248 (51%)	1,667 (51%)
**Male**	4,682 (48%)	681 (47%)	1,251 (48%)	1,175 (48%)	1,575 (48%)	4,742 (49%)	699 (49%)	1,279 (49%)	1,183 (49%)	1,581 (49%)
**Age**										
[**18,25**)	1,305 (13%)	187 (13%)	346 (13%)	294 (12%)	478 (15%)	1,197 (12%)	170 (12%)	339 (13%)	289 (12%)	400 (12%)
[**25,35**)	1,734 (18%)	280 (19%)	469 (18%)	449 (18%)	536 (17%)	1,681 (17%)	277 (19%)	445 (17%)	406 (17%)	553 (17%)
[**35,45**)	2,305 (24%)	300 (21%)	557 (21%)	559 (23%)	889 (27%)	1,723 (18%)	242 (17%)	451 (17%)	445 (18%)	584 (18%)
[**45,65**)	3,018 (31%)	456 (32%)	861 (33%)	770 (32%)	931 (29%)	3,111 (32%)	466 (32%)	865 (33%)	760 (31%)	1,020 (31%)
[**65,100**]	1,381 (14%)	214 (15%)	394 (15%)	359 (15%)	414 (13%)	2,030 (21%)	281 (20%)	528 (20%)	530 (22%)	691 (21%)
**City**										
**National**	4,677 (48%)	644 (45%)	1,392 (53%)	1,127 (46%)	1,514 (47%)	4,991 (51%)	685 (48%)	1,528 (58%)	1,171 (48%)	1,607 (49%)
**Atlanta**	916 (9.4%)	210 (15%)	203 (7.7%)	215 (8.8%)	288 (8.9%)	900 (9.2%)	217 (15%)	191 (7.3%)	236 (9.7%)	256 (7.9%)
**Bay Area**	820 (8.4%)	133 (9.3%)	206 (7.8%)	225 (9.3%)	256 (7.9%)	694 (7.1%)	122 (8.5%)	151 (5.8%)	184 (7.6%)	237 (7.3%)
**Boston**	866 (8.9%)	158 (11%)	199 (7.6%)	212 (8.7%)	297 (9.1%)	873 (9.0%)	158 (11%)	188 (7.2%)	215 (8.9%)	311 (9.6%)
**NY**	901 (9.2%)	150 (10%)	213 (8.1%)	224 (9.2%)	314 (9.7%)	752 (7.7%)	131 (9.1%)	189 (7.2%)	185 (7.6%)	246 (7.6%)
**Philadelphia**	723 (7.4%)	0 (0%)	219 (8.3%)	216 (8.9%)	288 (8.9%)	816 (8.4%)	0 (0%)	226 (8.6%)	249 (10%)	342 (11%)
**Phoenix**	840 (8.6%)	142 (9.9%)	195 (7.4%)	212 (8.7%)	291 (9.0%)	717 (7.4%)	124 (8.7%)	153 (5.8%)	190 (7.8%)	250 (7.7%)
**Urban/Rural**										
**Urban**	4,001 (42%)	578 (41%)	1,021 (39%)	988 (41%)	1,414 (45%)	2,958 (31%)	436 (31%)	798 (31%)	738 (31%)	986 (31%)
**Suburban**	4,928 (51%)	733 (52%)	1,409 (54%)	1,254 (52%)	1,532 (48%)	5,307 (55%)	783 (55%)	1,431 (55%)	1,324 (55%)	1,769 (55%)
**Rural**	663 (6.9%)	104 (7.3%)	169 (6.5%)	159 (6.6%)	231 (7.3%)	1,429 (15%)	211 (15%)	385 (15%)	356 (15%)	476 (15%)
**Ethnicity**										
**White**	7,166 (74%)	1,044 (73%)	1,942 (74%)	1,782 (73%)	2,398 (74%)	7,143 (73%)	1,059 (74%)	1,917 (73%)	1,794 (74%)	2,374 (73%)
**Black**	1,074 (11%)	174 (12%)	292 (11%)	247 (10%)	361 (11%)	1,147 (12%)	167 (12%)	308 (12%)	301 (12%)	371 (11%)
**Other**	1,503 (15%)	219 (15%)	393 (15%)	402 (17%)	489 (15%)	1,453 (15%)	211 (15%)	402 (15%)	337 (14%)	504 (16%)
**Hispanic**	935 (9.8%)	133 (9.4%)	273 (11%)	242 (10%)	287 (9.0%)	1,575 (16%)	232 (16%)	424 (16%)	393 (16%)	526 (16%)
**Education**										
**College graduate**	4,947 (51%)	667 (47%)	1,300 (50%)	1,245 (51%)	1,735 (54%)	2,925 (30%)	431 (30%)	789 (30%)	729 (30%)	975 (30%)
**Some college**	2,783 (29%)	452 (32%)	813 (31%)	651 (27%)	867 (27%)	2,327 (24%)	343 (24%)	628 (24%)	580 (24%)	776 (24%)
**High school graduate**	1,478 (15%)	249 (17%)	400 (15%)	339 (14%)	490 (15%)	3,507 (36%)	517 (36%)	946 (36%)	875 (36%)	1,169 (36%)
**Non-high school graduate**	480 (5.0%)	62 (4.3%)	106 (4.0%)	185 (7.6%)	127 (3.9%)	960 (9.9%)	142 (9.9%)	259 (9.9%)	240 (9.9%)	320 (9.9%)
**Household Size**										
**1**	1,768 (18%)	284 (20%)	516 (20%)	443 (18%)	525 (16%)	1,609 (17%)	237 (17%)	434 (17%)	402 (17%)	537 (17%)
**2**	2,955 (30%)	437 (30%)	822 (31%)	798 (33%)	898 (28%)	3,196 (33%)	471 (33%)	862 (33%)	798 (33%)	1,065 (33%)
**3**	2,031 (21%)	311 (22%)	526 (20%)	481 (20%)	713 (22%)	1,848 (19%)	273 (19%)	498 (19%)	461 (19%)	616 (19%)
**4**	2,024 (21%)	246 (17%)	513 (20%)	485 (20%)	780 (24%)	1,583 (16%)	233 (16%)	427 (16%)	395 (16%)	528 (16%)
**5 or more**	965 (9.9%)	159 (11%)	250 (9.5%)	224 (9.2%)	332 (10%)	1,506 (15%)	222 (15%)	406 (15%)	376 (15%)	502 (15%)
**Weekday**	7,100 (73%)	702 (49%)	1,918 (73%)	1,719 (71%)	2,761 (85%)	7,143 (73%)	684 (48%)	1,916 (73%)	1,768 (73%)	2,775 (85%)

[1] Statistics presented: n (%)

Supplementary note 1: City samples

For our city-specific samples, we recruited respondents who lived in the Designated Market Area (DMA) surrounding each city. DMAs are intended to capture media markets, and therefore often include much more than just the urban core of a city.

Our city-specific samples recruited respondents in the DMAs associated with six cities: Atlanta, the San Francisco Bay Area, Boston, New York, Phoenix and, starting in Wave 1, Philadelphia. Supplementary figure 1 shows the media markets we oversampled.

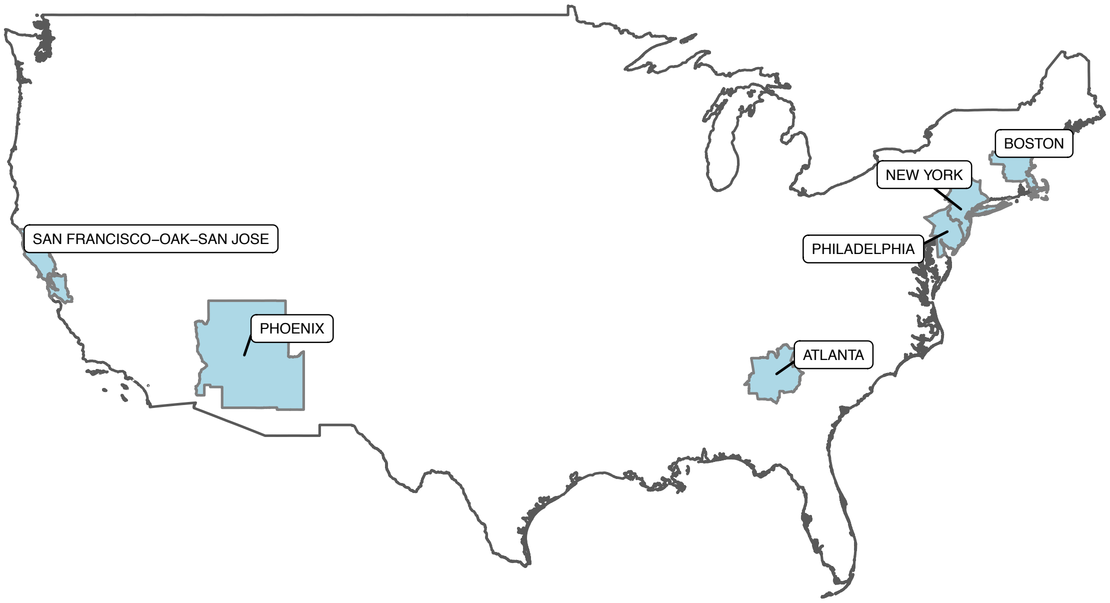

Supplementary figure 1: The geographical areas corresponding to the cities in our sample. Map created using data from Sood.[23]

Supplementary note 2: Comparison data from 2015 Facebook survey

The pre-pandemic baseline contact estimates come from a survey conducted among a random sample of Facebook users. The study interviewed 4,288 respondents in the United States; respondents were randomized to report about one of two different types of contact: either conversational contact or sharing a meal. Here, we focus on the results for conversational contacts, which is comparable to the contact we ask about in the BICS study. The sampling frame for survey respondents was people who use Facebook; a more detailed discussion of the study design can be found in the original paper.[9] Figure 4 compares, for a given age group, contact rates among people who used Facebook in 2015 and people who we contacted through the online panel in 2020. We expect these two groups – people who use Facebook and people reached by the online panel – to be very similar; however, if there are differences in contact rates between these two groups, then the estimates in Figure 4 could be affected. Note that, as Figure 4 shows, our

substantive conclusions are robust to using POLYMOD data from the UK as an alternate to the Facebook survey as a baseline.

Supplementary note 3: Bootstraped contact matrices

Uncertainty estimates for the descriptive results – including Figure 1 and the R_0 analysis summarized in Figure 4 – are based on the bootstrap. To obtain bootstrap resamples, we resampled respondents separately in each stratum (i.e., in each city and in the national sample). For each bootstrap resample, we first drew n_c samples with replacement from among the n_c respondents in city c. For resampled respondents who provided detailed reports about all contacts (i.e., those respondents who had $a_i = 1$ for all contacts i), we used the reported detailed contacts without resampling. For resampled respondents who did not provide detailed reports about all contacts, but who reported about a subset of contacts (i.e., for each respondent who had $a_i > 1$ for some contact i), we resampled r_i contacts with replacement from among the respondent's r_i detailed contacts. This second stage is intended to capture sampling variation due to the respondent choosing which contacts to report about from among her total contacts. Using this approach, we obtained 5,000 bootstrap resamples of our dataset, and these bootstrap resamples are the basis for uncertainty estimates.

Supplementary note 4: Average number of reported contacts

Here, we present additional analyses of reported contacts. First, Supplementary table 2 shows the average number of reported contacts for several different groups of respondents, and Supplementary table 3 shows analagous numbers without using the weights. Note that these averages are based on topcoded values.

Supplementary table 2: Average number of contacts and non-household contacts, by survey wave.

	All contacts, weighted				Non-household contacts, weighted			
	Wave 0	Wave 1	Wave 2	Wave 3	Wave 0	Wave 1	Wave 2	Wave 3
**Location**								
**(Unknown)**	2.8 (2.1, 3.5)	7.6 (3.6, 13.4)	5.1 (3.6, 7.3)	6.3 (4.0, 9.2)	0.8 (0.4, 1.4)	5.4 (1.7, 11.5)	2.2 (0.8, 4.4)	4.2 (2.2, 7.0)
**Rural**	2.7 (2.2, 3.3)	3.6 (2.8, 4.6)	4.9 (4.0, 6.1)	9.1 (6.2, 12.6)	1.2 (0.7, 1.8)	2.0 (1.3, 2.9)	3.2 (2.3, 4.4)	7.1 (4.3, 10.6)
**Suburban**	2.8 (2.6, 3.0)	4.2 (3.8, 4.7)	5.1 (4.6, 5.7)	6.2 (5.6, 6.9)	1.3 (1.1, 1.5)	2.3 (1.9, 2.8)	3.3 (2.8, 3.8)	4.4 (3.8, 5.1)
**Urban**	2.8 (2.6, 3.0)	4.2 (3.8, 4.6)	5.3 (4.7, 5.8)	5.5 (5.1, 6.0)	1.4 (1.2, 1.6)	2.3 (1.9, 2.6)	3.3 (2.8, 3.9)	3.6 (3.1, 4.0)
**Age**								
**[18,25)**	3.4 (2.9, 3.9)	6.1 (4.7, 7.7)	5.4 (4.7, 6.3)	6.8 (5.7, 8.0)	1.5 (1.0, 2.2)	3.6 (2.3, 5.2)	2.7 (2.0, 3.5)	4.2 (3.1, 5.4)
**[25,35)**	3.1 (2.7, 3.4)	4.4 (3.9, 4.9)	6.4 (5.3, 7.7)	7.4 (6.2, 8.8)	1.6 (1.2, 2.0)	2.2 (1.7, 2.7)	4.3 (3.3, 5.6)	5.3 (4.1, 6.7)
**[35,45)**	3.2 (2.9, 3.5)	5.1 (4.6, 5.7)	6.0 (5.3, 6.8)	7.9 (6.9, 9.1)	1.4 (1.2, 1.7)	2.7 (2.2, 3.2)	3.6 (2.9, 4.4)	5.6 (4.6, 6.7)
**[45,65)**	2.6 (2.4, 2.9)	3.8 (3.3, 4.3)	5.2 (4.5, 5.9)	6.9 (5.5, 8.6)	1.2 (1.0, 1.4)	2.1 (1.7, 2.6)	3.5 (2.8, 4.2)	5.2 (3.8, 6.9)
**[65,100]**	2.0 (1.7, 2.3)	2.5 (2.1, 3.0)	3.3 (2.8, 3.8)	3.4 (2.8, 4.1)	1.0 (0.8, 1.3)	1.5 (1.1, 1.9)	2.2 (1.8, 2.7)	2.4 (1.8, 3.0)
**Race/Ethnicity**								
**(Unknown)**	1.9 (1.0, 3.0)	5.6 (3.5, 7.8)	4.6 (3.4, 6.1)	4.5 (2.6, 7.7)	0.7 (0.2, 1.5)	3.1 (1.4, 5.2)	2.8 (1.7, 4.1)	3.3 (1.5, 6.3)
**Black, non-Hispanic**	2.7 (2.2, 3.2)	4.5 (3.5, 5.8)	4.8 (3.9, 6.0)	5.1 (4.1, 6.4)	1.3 (1.0, 1.7)	3.0 (2.0, 4.3)	3.1 (2.2, 4.3)	3.6 (2.6, 4.8)
**Hispanic**	3.0 (2.6, 3.5)	4.7 (4.0, 5.6)	5.9 (5.0, 7.0)	5.5 (4.7, 6.6)	1.5 (1.0, 2.1)	2.4 (1.7, 3.2)	3.5 (2.6, 4.6)	3.2 (2.3, 4.2)
**Other, non-Hispanic**	2.5 (2.2, 2.9)	3.9 (3.1, 4.9)	4.4 (3.7, 5.2)	5.0 (4.2, 5.9)	1.0 (0.7, 1.3)	1.7 (1.0, 2.7)	2.2 (1.6, 2.9)	3.2 (2.4, 4.0)
**White, non-Hispanic**	2.8 (2.6, 2.9)	4.0 (3.7, 4.4)	5.1 (4.6, 5.5)	7.0 (6.2, 7.9)	1.3 (1.1, 1.5)	2.2 (1.9, 2.6)	3.3 (2.9, 3.8)	5.2 (4.4, 6.1)
**Day of week**								
**Weekday**	2.8 (2.6, 3.0)	4.1 (3.8, 4.5)	5.1 (4.7, 5.6)	6.6 (6.0, 7.3)	1.3 (1.2, 1.5)	2.2 (1.9, 2.6)	3.2 (2.8, 3.6)	4.7 (4.1, 5.4)
**Weekend**	2.7 (2.5, 2.9)	4.3 (3.7, 5.0)	5.2 (4.6, 5.9)	5.5 (4.4, 7.0)	1.3 (1.1, 1.5)	2.4 (1.9, 3.1)	3.5 (2.9, 4.1)	3.8 (2.7, 5.3)
**Education**								
**(Unknown)**	5.1 (1.9, 8.0)	3.3 (1.6, 4.8)	4.6 (0.8, 12.4)	2.3 (1.8, 3.1)	3.4 (0.6, 6.4)	1.5 (0.5, 2.6)	4.0 (0.1, 11.5)	1.1 (0.8, 1.5)
**College graduate**	2.8 (2.6, 3.0)	4.4 (4.1, 4.8)	5.3 (4.8, 5.8)	6.7 (6.3, 7.2)	1.4 (1.2, 1.6)	2.5 (2.1, 2.8)	3.4 (3.0, 3.9)	4.8 (4.3, 5.2)
**College, non-graduate**	2.8 (2.5, 3.0)	4.2 (3.6, 4.8)	5.4 (4.7, 6.1)	5.8 (5.1, 6.7)	1.3 (1.1, 1.5)	2.3 (1.8, 2.9)	3.5 (2.9, 4.3)	4.0 (3.3, 4.9)
**HS graduate**	2.6 (2.3, 2.9)	3.7 (3.3, 4.3)	4.8 (4.1, 5.5)	6.3 (4.9, 7.9)	1.1 (0.9, 1.4)	2.0 (1.5, 2.5)	3.0 (2.3, 3.7)	4.6 (3.2, 6.1)
**HS, non-graduate**	3.3 (2.6, 4.0)	4.9 (3.5, 6.7)	5.5 (4.7, 6.4)	7.4 (5.5, 9.5)	1.6 (0.9, 2.4)	2.8 (1.5, 4.5)	3.2 (2.4, 4.1)	5.3 (3.5, 7.4)
**Gender**								
**Female**	2.8 (2.6, 3.0)	3.9 (3.5, 4.3)	4.9 (4.4, 5.4)	6.2 (5.4, 7.2)	1.2 (1.0, 1.4)	2.0 (1.6, 2.4)	3.0 (2.6, 3.5)	4.4 (3.6, 5.4)
**Male**	2.8 (2.6, 3.0)	4.5 (4.0, 5.0)	5.4 (4.9, 6.0)	6.6 (5.9, 7.5)	1.4 (1.2, 1.7)	2.6 (2.2, 3.1)	3.6 (3.1, 4.1)	4.7 (4.0, 5.6)
**Household size**								
**1**	1.8 (1.5, 2.1)	2.4 (1.7, 3.2)	3.1 (2.4, 4.0)	4.4 (2.6, 6.9)	1.4 (1.2, 1.7)	2.4 (1.7, 3.2)	3.1 (2.4, 4.0)	4.4 (2.6, 6.9)
**2**	2.0 (1.8, 2.2)	2.8 (2.5, 3.1)	4.0 (3.5, 4.6)	4.8 (4.0, 5.5)	1.0 (0.8, 1.2)	1.8 (1.5, 2.1)	3.0 (2.5, 3.6)	3.8 (3.0, 4.5)
**3**	2.8 (2.6, 3.1)	4.4 (3.8, 5.2)	5.4 (4.7, 6.3)	7.0 (5.9, 8.4)	1.2 (1.0, 1.5)	2.4 (1.8, 3.2)	3.4 (2.7, 4.3)	5.0 (3.9, 6.4)
**4**	3.7 (3.3, 4.0)	5.5 (4.9, 6.2)	6.7 (5.9, 7.6)	8.2 (7.2, 9.3)	1.2 (0.9, 1.5)	2.5 (1.9, 3.2)	3.7 (2.9, 4.6)	5.2 (4.2, 6.3)
**5 or more**	4.6 (4.1, 5.1)	7.3 (6.3, 8.4)	7.7 (6.8, 8.9)	9.5 (7.8, 11.7)	2.2 (1.6, 2.8)	2.9 (1.9, 4.0)	3.4 (2.4, 4.5)	5.2 (3.5, 7.3)

Supplementary table 3: Average number of contacts and non-household contacts, by survey wave.

	All contacts, unweighted				Non-household contacts, unweighted			
	Wave 0	Wave 1	Wave 2	Wave 3	Wave 0	Wave 1	Wave 2	Wave 3
**Location**								
**(Unknown)**	2.7 (2.0, 3.3)	6.3 (3.4, 10.7)	5.9 (3.7, 9.0)	7.5 (4.6, 11.3)	1.0 (0.6, 1.6)	4.4 (1.5, 8.9)	3.5 (1.2, 6.6)	5.5 (2.5, 9.3)
**Rural**	2.6 (2.2, 3.1)	3.8 (3.0, 4.6)	5.3 (4.2, 6.5)	8.5 (6.7, 10.6)	1.2 (0.8, 1.5)	2.3 (1.6, 3.1)	3.7 (2.7, 4.9)	6.5 (4.7, 8.6)
**Suburban**	2.7 (2.5, 2.8)	4.1 (3.7, 4.4)	5.2 (4.7, 5.6)	6.6 (6.1, 7.3)	1.2 (1.1, 1.3)	2.3 (2.0, 2.7)	3.5 (3.1, 4.0)	4.9 (4.3, 5.5)
**Urban**	2.7 (2.5, 2.9)	4.3 (3.9, 4.8)	5.3 (4.8, 5.9)	6.4 (5.9, 7.0)	1.4 (1.3, 1.6)	2.6 (2.2, 3.1)	3.5 (3.0, 4.1)	4.5 (4.0, 5.0)
**Age**								
**[18,25)**	3.0 (2.7, 3.3)	5.2 (4.3, 6.2)	5.5 (4.6, 6.5)	6.4 (5.5, 7.5)	1.1 (0.9, 1.3)	2.9 (2.1, 4.0)	3.1 (2.3, 4.1)	4.3 (3.4, 5.3)
**[25,35)**	2.8 (2.6, 3.1)	4.2 (3.7, 4.8)	5.6 (4.9, 6.4)	7.4 (6.5, 8.4)	1.5 (1.2, 1.8)	2.4 (1.9, 3.0)	3.8 (3.1, 4.6)	5.5 (4.6, 6.5)
**[35,45)**	3.1 (2.9, 3.3)	5.3 (4.7, 6.0)	6.0 (5.4, 6.6)	8.2 (7.5, 9.0)	1.5 (1.3, 1.8)	3.1 (2.5, 3.8)	3.8 (3.2, 4.4)	5.9 (5.2, 6.7)
**[45,65)**	2.6 (2.4, 2.7)	3.6 (3.3, 4.1)	5.2 (4.5, 6.0)	6.3 (5.6, 7.1)	1.2 (1.1, 1.4)	2.1 (1.8, 2.6)	3.7 (3.0, 4.5)	4.7 (3.9, 5.5)
**[65,100]**	2.0 (1.8, 2.3)	2.8 (2.2, 3.5)	3.6 (3.0, 4.3)	3.6 (2.9, 4.5)	1.0 (0.8, 1.2)	1.8 (1.3, 2.5)	2.6 (2.0, 3.3)	2.6 (2.0, 3.5)
**Race/Ethnicity**								
**(Unknown)**	2.4 (1.4, 3.6)	5.1 (3.2, 7.3)	4.4 (3.4, 5.5)	5.3 (2.8, 9.4)	1.1 (0.3, 2.1)	3.0 (1.3, 5.2)	2.7 (1.9, 3.7)	4.2 (1.8, 8.2)
**Black, non-Hispanic**	2.4 (2.1, 2.7)	4.4 (3.6, 5.2)	4.7 (3.8, 5.8)	5.2 (4.3, 6.3)	1.2 (0.9, 1.5)	2.9 (2.2, 3.8)	3.2 (2.2, 4.3)	3.6 (2.7, 4.6)
**Hispanic**	2.9 (2.5, 3.3)	4.6 (4.0, 5.5)	5.7 (4.8, 6.7)	5.3 (4.6, 6.0)	1.5 (1.1, 1.8)	2.7 (2.0, 3.5)	3.7 (2.8, 4.8)	3.1 (2.5, 3.9)
**Other, non-Hispanic**	2.4 (2.1, 2.6)	3.5 (2.7, 4.6)	4.1 (3.4, 5.1)	5.0 (4.2, 5.8)	0.8 (0.6, 1.1)	1.7 (0.9, 2.8)	2.2 (1.5, 3.2)	3.2 (2.5, 4.0)
**White, non-Hispanic**	2.8 (2.6, 2.9)	4.2 (3.8, 4.5)	5.5 (5.0, 5.9)	7.4 (6.9, 7.9)	1.4 (1.2, 1.5)	2.4 (2.1, 2.8)	3.8 (3.3, 4.2)	5.5 (5.0, 6.0)
**Day of week**								
**Weekday**	2.7 (2.6, 2.9)	4.1 (3.8, 4.4)	5.2 (4.8, 5.6)	6.9 (6.5, 7.3)	1.4 (1.2, 1.5)	2.4 (2.1, 2.7)	3.4 (3.1, 3.8)	5.0 (4.6, 5.4)
**Weekend**	2.7 (2.5, 2.8)	4.3 (3.8, 4.9)	5.4 (4.7, 6.1)	5.6 (4.8, 6.5)	1.2 (1.1, 1.4)	2.6 (2.1, 3.1)	3.7 (3.1, 4.5)	3.8 (3.1, 4.7)
**Education**								
**(Unknown)**	4.6 (1.8, 7.5)	3.1 (1.4, 4.9)	3.9 (1.0, 8.7)	3.0 (2.0, 4.2)	2.9 (0.5, 5.8)	1.5 (0.5, 2.8)	2.9 (0.3, 7.5)	1.8 (1.0, 3.0)
**College graduate**	2.7 (2.5, 2.9)	4.3 (4.0, 4.7)	5.4 (4.9, 5.9)	7.3 (6.8, 7.9)	1.4 (1.2, 1.5)	2.5 (2.2, 2.9)	3.7 (3.2, 4.2)	5.3 (4.8, 5.9)
**College, non-graduate**	2.6 (2.4, 2.8)	4.0 (3.6, 4.5)	5.3 (4.6, 6.1)	6.0 (5.3, 6.8)	1.2 (1.1, 1.4)	2.4 (1.9, 2.9)	3.6 (2.9, 4.4)	4.3 (3.5, 5.0)
**HS graduate**	2.6 (2.4, 2.9)	3.9 (3.3, 4.7)	4.6 (3.9, 5.3)	6.0 (5.0, 7.1)	1.1 (0.9, 1.4)	2.2 (1.6, 3.0)	3.0 (2.3, 3.7)	4.3 (3.3, 5.4)
**HS, non-graduate**	3.0 (2.5, 3.5)	4.7 (3.5, 6.4)	5.2 (4.4, 6.2)	6.6 (5.0, 8.4)	1.3 (0.8, 1.8)	2.9 (1.6, 4.5)	3.1 (2.3, 4.1)	4.7 (3.2, 6.4)
**Gender**								
**Female**	2.6 (2.5, 2.8)	3.8 (3.5, 4.2)	5.0 (4.6, 5.5)	6.3 (5.7, 6.9)	1.1 (1.0, 1.3)	2.1 (1.8, 2.5)	3.3 (2.9, 3.8)	4.5 (4.0, 5.1)
**Male**	2.8 (2.6, 2.9)	4.6 (4.2, 5.0)	5.5 (5.0, 6.0)	7.1 (6.6, 7.7)	1.5 (1.3, 1.6)	2.8 (2.4, 3.2)	3.8 (3.3, 4.3)	5.2 (4.6, 5.7)
**Household size**								
**1**	1.7 (1.5, 1.9)	2.5 (2.0, 3.2)	3.6 (2.8, 4.7)	4.2 (3.3, 5.3)	1.4 (1.2, 1.7)	2.5 (2.0, 3.2)	3.6 (2.8, 4.7)	4.2 (3.3, 5.3)
**2**	2.0 (1.8, 2.1)	3.0 (2.7, 3.4)	4.1 (3.6, 4.7)	4.6 (4.0, 5.3)	1.0 (0.8, 1.1)	2.0 (1.7, 2.4)	3.1 (2.6, 3.7)	3.6 (3.0, 4.3)
**3**	2.9 (2.7, 3.1)	4.7 (4.1, 5.4)	5.6 (4.9, 6.3)	7.3 (6.6, 8.1)	1.3 (1.1, 1.6)	2.7 (2.1, 3.4)	3.6 (2.9, 4.3)	5.3 (4.6, 6.1)
**4**	3.6 (3.4, 3.8)	5.7 (5.1, 6.4)	7.2 (6.4, 8.1)	9.0 (8.1, 9.9)	1.2 (1.0, 1.5)	2.7 (2.1, 3.4)	4.2 (3.4, 5.1)	6.0 (5.1, 6.9)
**5 or more**	4.7 (4.3, 5.0)	7.1 (6.3, 8.2)	7.6 (6.8, 8.4)	9.7 (8.5, 11.2)	2.0 (1.6, 2.4)	2.7 (1.9, 3.8)	3.3 (2.5, 4.1)	5.4 (4.2, 6.9)

Next, in addition to the model fit to non-household contacts (Figure 2), we also fit a model to all contacts; Supplementary figure 2 shows predictions from this model. The figure suggests that inferences from this model fit to all contacts are qualitatively very similar to the ones from the model fit to non-household contacts (Figure 2); the primary difference is in the estimated effect of household size. To confirm this finding, Supplementary figure 3 directly compares estimated coefficients from the two models. The figure shows a very strong positive relationship for all predictors, except for household size. The discrepancy between coefficient estimates for household size can be explained by the fact that household members are counted in all contacts, meaning there is a direct relationship between household size and all contacts.

Predictions come from a negative binomial model fit to reported numbers of non-household contacts made by $n = 9,743$ survey respondents.

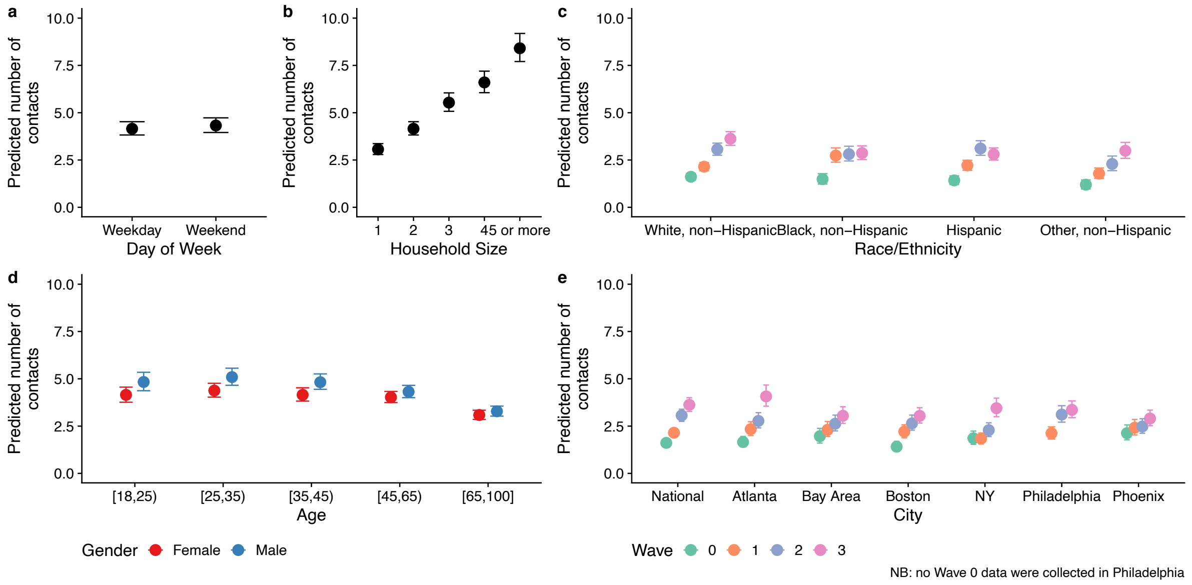

Supplementary figure 2: Conditional effect plots showing the predicted mean number of contacts and 95% posterior credible intervals for several covariates. Predicted mean number of contacts shown for (A) day of the week; (B) Household size; (C) Race/ethnicity; (D) Age/sex group; and (E) Geography. Predictions come from a negative binomial model fit to reported numbers of all contacts made by $n = 9,743$ survey respondents. Colors are used in panels C, D, and E to show estimated interactions. Covariate values not being manipulated in each panel are set to values for a white female aged 35-44 from the national sample who lives in a two-person household during a weekday in wave 3 (Methods). Uncertainty bars show 95% posterior credible intervals. Figure 2 shows the same predictions for an analogous model fit to non-household contacts.

Finally, Supplementary table 4 reports posterior mean estimates, estimated standard errors, and 95% credible intervals for coefficients from the two models. Note that Figures 2 and 2 illustrate these estimates in a more interpretable way.

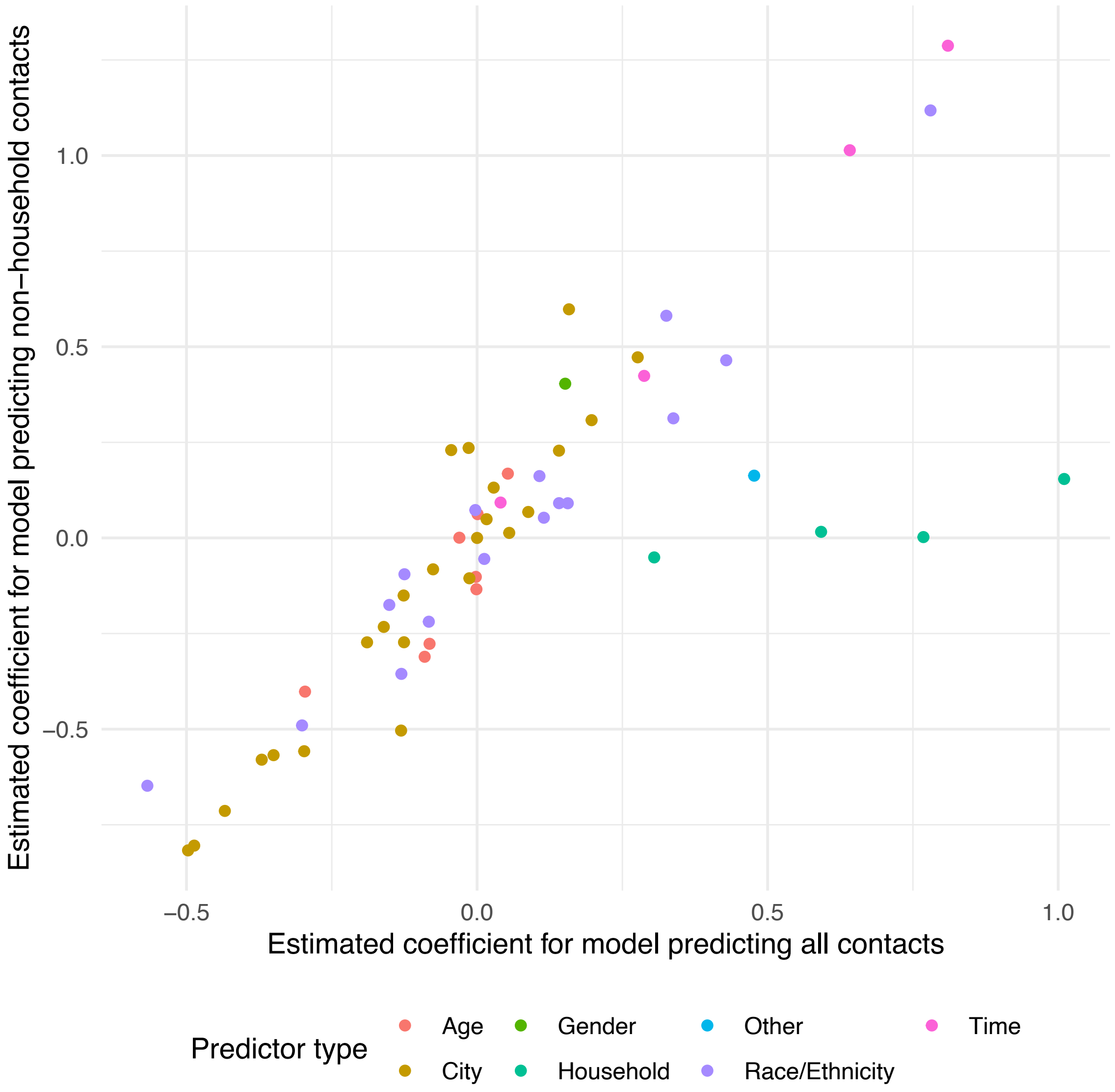

Supplementary figure 3: Comparison between coefficients on different predictors for a negative binomial regression model predicting all contacts (x axis, see also Supplementary figure 2) and for an analogous negative binomial regression model predicting non-household contacts (y axis, see also Figure 2). There is a strong, positive relationship in estimated coefficients across the two models for all predictors except for household size.

Supplementary table 4: Posterior mean estimates, estimated standard errors, and 95% posterior credible intervals for coefficients in regression models predicting all contacts and non-household contacts. Reference categories are shown with dashes.

		All contacts				Non-household contacts			
	Predictor value	Estimate	Std. Err.	Lower CI	Upper CI	Estimate	Std. Err.	Lower CI	Upper CI
**Age**	18-25	-	-	-	-	-	-	-	-
	25-35	0.05	0.05	-0.04	0.14	0.17	0.09	-0.01	0.33
	25-35 X Male	0.00	0.07	-0.13	0.13	-0.13	0.12	-0.37	0.10
	35-45	0.00	0.05	-0.09	0.09	0.06	0.09	-0.11	0.23
	35-45 X Male	0.00	0.07	-0.13	0.12	-0.10	0.12	-0.33	0.14
	45-65	-0.03	0.04	-0.12	0.05	0.00	0.08	-0.16	0.15
	45-65 X Male	-0.08	0.06	-0.20	0.03	-0.28	0.11	-0.48	-0.07
	65-100	-0.30	0.05	-0.39	-0.20	-0.40	0.09	-0.57	-0.23
	65-100 X Male	-0.09	0.07	-0.22	0.04	-0.31	0.12	-0.54	-0.08
	Atlanta	0.03	0.08	-0.11	0.18	0.13	0.14	-0.12	0.40
	BayArea	0.20	0.09	0.01	0.38	0.31	0.17	-0.01	0.66
	Boston	-0.13	0.09	-0.30	0.04	-0.50	0.16	-0.83	-0.19
	National	-	-	-	-	-	-	-	-
	NY	0.14	0.09	-0.04	0.32	0.23	0.16	-0.08	0.55
	Philadelphia	0.00	0.00	0.00	0.00	0.00	0.00	0.00	0.00
	Phoenix	0.28	0.09	0.10	0.45	0.47	0.16	0.15	0.78
	Wave 1 X Atlanta	0.06	0.10	-0.14	0.25	0.01	0.19	-0.34	0.37
	Wave 1 X BayArea	-0.13	0.12	-0.36	0.12	-0.15	0.22	-0.59	0.27
	Wave 1 X Boston	0.16	0.11	-0.06	0.37	0.60	0.20	0.20	1.00
	Wave 1 X NY	-0.30	0.12	-0.52	-0.07	-0.56	0.20	-0.97	-0.17
	Wave 1 X Philadelphia	-0.01	0.06	-0.14	0.11	-0.11	0.12	-0.34	0.12

City

Supplementary table 4: Posterior mean estimates, estimated standard errors, and 95% posterior credible intervals for coefficients in regression models predicting all contacts and non-household contacts. Reference categories are shown with dashes. *(continued)*

	Predictor value	Estimate	Std. Err.	Lower CI	Upper CI	Estimate	Std. Err.	Lower CI	Upper CI
	Wave 1 X Phoenix	-0.16	0.11	-0.38	0.06	-0.23	0.21	-0.64	0.19
	Wave 2 X Atlanta	-0.13	0.10	-0.32	0.06	-0.27	0.18	-0.62	0.07
	Wave 2 X BayArea	-0.35	0.12	-0.58	-0.12	-0.57	0.21	-1.00	-0.16
	Wave 2 X Boston	-0.01	0.11	-0.22	0.19	0.24	0.20	-0.16	0.64
	Wave 2 X NY	-0.43	0.12	-0.66	-0.20	-0.71	0.20	-1.12	-0.32
	Wave 2 X Philadelphia	0.02	0.06	-0.10	0.14	0.05	0.11	-0.16	0.27
	Wave 2 X Phoenix	-0.49	0.11	-0.70	-0.27	-0.80	0.20	-1.21	-0.42
	Wave 3 X Atlanta	0.09	0.09	-0.10	0.27	0.07	0.17	-0.27	0.40
	Wave 3 X BayArea	-0.37	0.11	-0.59	-0.14	-0.58	0.20	-0.98	-0.20
	Wave 3 X Boston	-0.04	0.10	-0.24	0.15	0.23	0.19	-0.14	0.60
	Wave 3 X NY	-0.19	0.11	-0.41	0.03	-0.27	0.19	-0.66	0.09
	Wave 3 X Philadelphia	-0.08	0.05	-0.18	0.02	-0.08	0.09	-0.26	0.10
	Wave 3 X Phoenix	-0.50	0.11	-0.70	-0.29	-0.82	0.20	-1.20	-0.42
**Gender**	Female	-	-	-	-	-	-	-	-
	Male	0.15	0.05	0.05	0.25	0.40	0.09	0.22	0.58
**Household**	HH size: 1	-	-	-	-	-	-	-	-
	HH size: 2	0.30	0.03	0.25	0.36	-0.05	0.05	-0.15	0.05
	HH size: 3	0.59	0.03	0.53	0.66	0.02	0.06	-0.10	0.13
	HH size: 4	0.77	0.03	0.70	0.84	0.00	0.06	-0.11	0.12
	HH size: 5ormore	1.01	0.03	0.94	1.08	0.15	0.06	0.04	0.27
**Other**	Intercept	0.48	0.06	0.36	0.59	0.16	0.10	-0.04	0.36
	Black, non-HispanicnonMHispanic	-0.08	0.09	-0.26	0.09	-0.22	0.15	-0.52	0.09

Supplementary table 4: Posterior mean estimates, estimated standard errors, and 95% posterior credible intervals for coefficients in regression models predicting all contacts and non-household contacts. Reference categories are shown with dashes. *(continued)*

	Predictor value	Estimate	Std. Err.	Lower CI	Upper CI	Estimate	Std. Err.	Lower CI	Upper CI
**Race/Ethnicity**	Black, non-HispanicnonMHispanic X Wave 1	0.33	0.11	0.12	0.53	0.58	0.19	0.20	0.94
	Black, non-HispanicnonMHispanic X Wave 2	0.00	0.10	-0.21	0.20	0.07	0.19	-0.31	0.44
	Black, non-HispanicnonMHispanic X Wave 3	-0.15	0.10	-0.35	0.05	-0.18	0.18	-0.53	0.18
	Hispanic	-0.12	0.07	-0.26	0.01	-0.09	0.13	-0.35	0.15
	Hispanic X Wave 1	0.16	0.08	-0.01	0.32	0.09	0.15	-0.22	0.40
	Hispanic X Wave 2	0.14	0.08	-0.02	0.30	0.09	0.15	-0.21	0.38
	Hispanic X Wave 3	-0.13	0.08	-0.29	0.03	-0.36	0.15	-0.64	-0.06
	Other, non-HispanicnonMHispanic	-0.30	0.09	-0.48	-0.12	-0.49	0.18	-0.83	-0.14
	Other, non-HispanicnonMHispanic X Wave 1	0.11	0.11	-0.11	0.34	0.05	0.21	-0.37	0.47
	Other, non-HispanicnonMHispanic X Wave 2	0.01	0.12	-0.21	0.25	-0.05	0.22	-0.48	0.37
	Other, non-HispanicnonMHispanic X Wave 3	0.11	0.11	-0.10	0.32	0.16	0.21	-0.24	0.56
	Unknown	-0.57	0.42	-1.40	0.23	-0.65	0.78	-2.14	0.92
	Unknown X Wave 1	0.78	0.47	-0.15	1.72	1.12	0.88	-0.66	2.87
	Unknown X Wave 2	0.43	0.49	-0.53	1.42	0.46	0.91	-1.35	2.23
	Unknown X Wave 3	0.34	0.49	-0.59	1.31	0.31	0.90	-1.49	2.02
	White, non-Hispanic	-	-	-	-	-	-	-	-
**Time**	Weekday	-	-	-	-	-	-	-	-
	Weekend	0.04	0.02	0.00	0.09	0.09	0.04	0.01	0.17
	Wave 0	-	-	-	-	-	-	-	-
	Wave 1	0.29	0.05	0.20	0.38	0.42	0.08	0.26	0.59
	Wave 2	0.64	0.05	0.55	0.74	1.01	0.09	0.84	1.18
	Wave 3	0.81	0.05	0.72	0.90	1.29	0.08	1.13	1.45

Supplementary note 5: Relationships and locations

Here, we present Tables containing the values shown in Figure 1, Panels C and D. These numerical values may be useful as numerical inputs for future modeling work.

Relationships

	Wave 0	Wave 1	Wave 2	Wave 3
Neighbor/community member	0.21 (0.16, 0.26)	0.22 (0.15, 0.33)	0.21 (0.14, 0.29)	0.34 (0.23, 0.49)
Spouse/romantic partner	0.04 (0.02, 0.07)	0.3 (0.2, 0.42)	0.25 (0.19, 0.33)	0.43 (0.34, 0.54)
Other	0.12 (0.08, 0.17)	0.25 (0.15, 0.39)	0.29 (0.21, 0.39)	0.4 (0.27, 0.56)
Work colleague/classmate	0.15 (0.1, 0.21)	0.45 (0.3, 0.65)	0.73 (0.54, 0.94)	1.35 (1, 1.76)
Friend	0.33 (0.27, 0.39)	0.58 (0.45, 0.75)	0.71 (0.58, 0.86)	0.89 (0.72, 1.08)
Family	0.23 (0.18, 0.28)	0.6 (0.47, 0.75)	0.78 (0.64, 0.94)	0.86 (0.67, 1.1)
I am this person's customer/client*		0.33 (0.24, 0.45)	0.38 (0.3, 0.47)	0.34 (0.27, 0.41)
This person is my customer/client*		0.13 (0.06, 0.24)	0.2 (0.09, 0.33)	0.47 (0.23, 0.83)

* These relationships were added to the instrument after Wave 0.

Locations

	Wave 0	Wave 1	Wave 2	Wave 3
Place of worship	0.01 (0, 0.03)	0.09 (0.02, 0.19)	0.08 (0.03, 0.15)	0.12 (0.05, 0.22)
Transit	0.04 (0.02, 0.06)	0.12 (0.05, 0.23)	0.07 (0.03, 0.11)	0.1 (0.06, 0.15)
School	0.04 (0.01, 0.1)	0.12 (0.05, 0.23)	0.06 (0.03, 0.11)	0.31 (0.18, 0.48)
Bar/Restaurant	0.03 (0.01, 0.06)	0.16 (0.08, 0.27)	0.23 (0.16, 0.32)	0.41 (0.25, 0.67)
Other	0.14 (0.09, 0.2)	0.17 (0.11, 0.26)	0.23 (0.16, 0.3)	0.28 (0.17, 0.42)
On the street	0.21 (0.16, 0.26)	0.24 (0.16, 0.35)	0.2 (0.14, 0.28)	0.23 (0.17, 0.31)
Store/Business	0.21 (0.15, 0.28)	0.6 (0.44, 0.81)	0.62 (0.48, 0.77)	0.62 (0.5, 0.74)
Work	0.17 (0.11, 0.24)	0.45 (0.29, 0.64)	0.81 (0.62, 1.04)	1.57 (1.15, 2.08)
Someone's home*		1.09 (0.9, 1.3)	1.3 (1.11, 1.5)	1.45 (1.24, 1.71)

* Wording for this response changed after Wave 0.

Supplementary note 6: Sensitivity analysis for contact definition

In Wave 0 (the pilot study), contact was defined using 'conversational contact', as explained in this text:

> By **in-person conversational contact**, we mean a two-way conversation with three or more words in the physical presence of another person.
>
> You might have conversational contact with family members, friends, co-workers, store clerks, bus drivers, and so forth.

> (**Please do not count people you contacted exclusively by telephone, text, or online.** Only consider people you interacted with face-to-face.)

After the pilot study, in Waves 1 and up, the survey instrument was modified and contact was defined using this text:

> Now we would like to ask you some questions about people you had **in-person contact** with yesterday.
>
> By **in-person contact**, we mean
>
> **EITHER** a **two-way conversation** with three or more words in the physical presence of another person **OR** physical **skin-to-skin contact** (for example a handshake, hug, kiss, or contact sports)
>
> You might have in-person contact with family members, friends, co-workers, store clerks, bus drivers, and so forth.
>
> **Please do not count people you contacted exclusively by telephone, text, or online**. Only consider people you interacted with face-to-face.

In the main text, for Waves 1 and up we combine physical and conversational contacts together, since the combination of these two is most relevant for disease transmission. However, just under 10 percent of non-household detailed contacts in Waves 1 and up were reported to be strictly physical (and not conversational). To assess how sensitive our results are to including all contacts in our analysis, we repeated key analyses excluding these contacts reported to be only physical in Waves 1 and up. Figures 4 and 5 suggest that this decision does not substantively affect our conclusions.

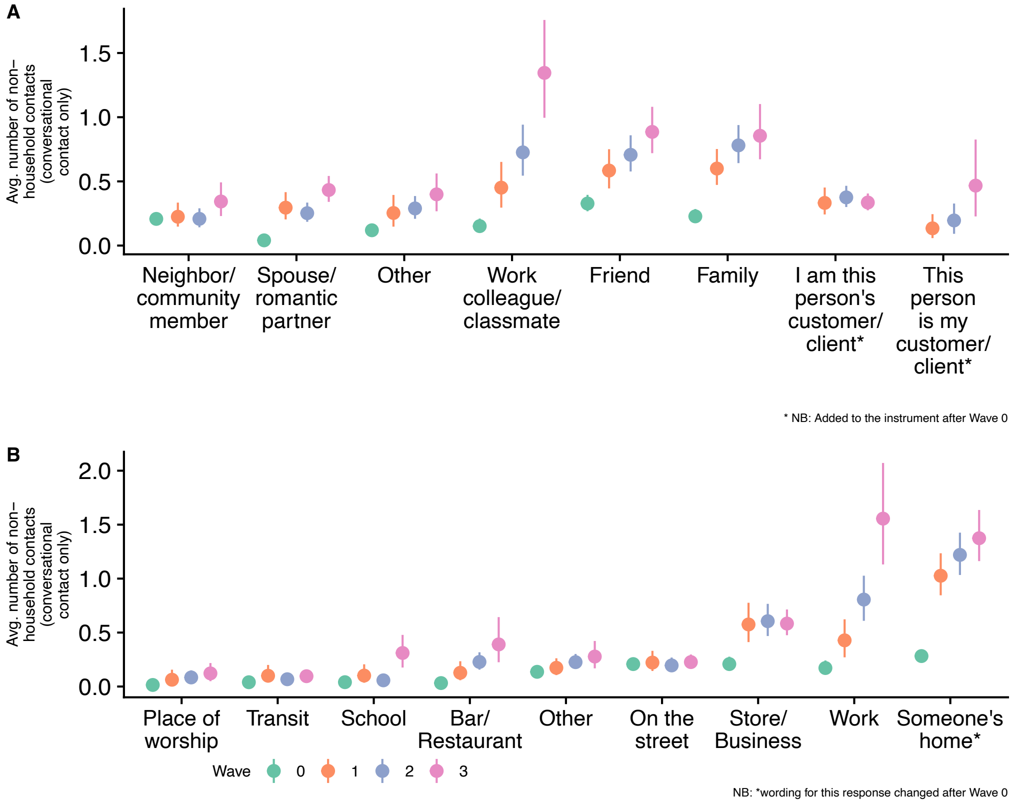

Supplementary figure 4: Estimated average number of contacts each person reported to have taken place by contact's relationship (top panel) and location (bottom panel). Uncertainty estimates are 95% intervals derived from the bootstrap. Contacts are restricted to those who were conversational contacts for Waves 1 and up. Estimates are based on $n = 29,880$ reports about detailed contacts (Methods).

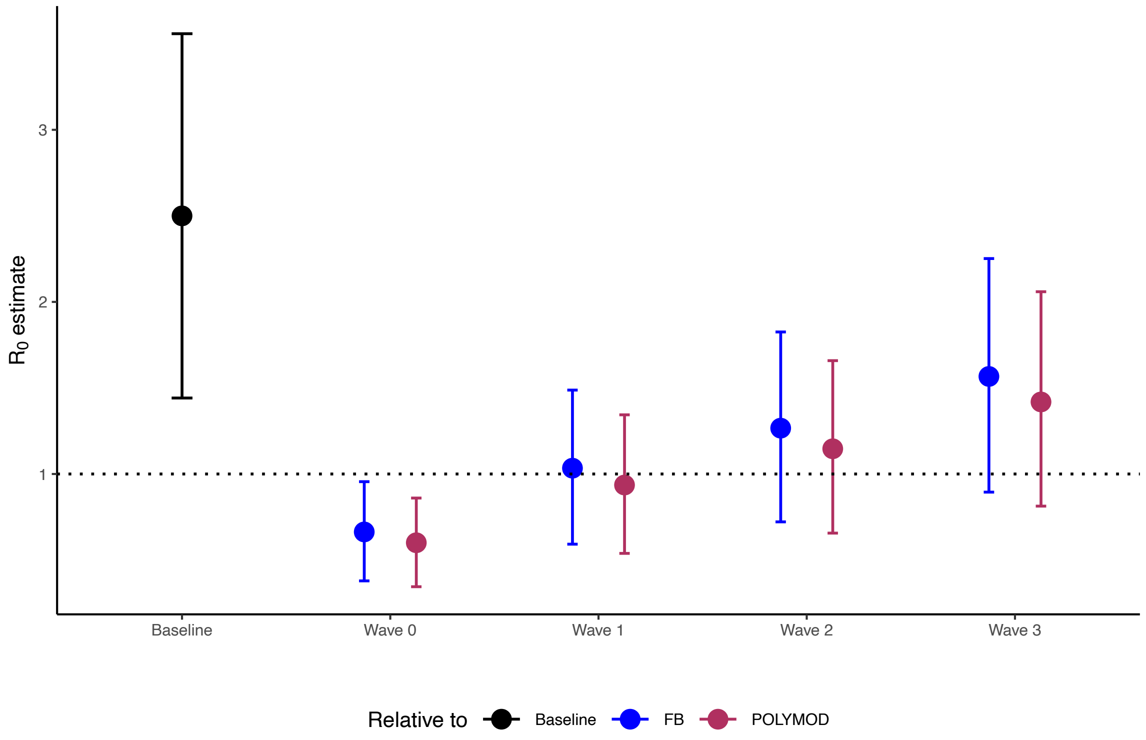

Supplementary figure 5: Implied R_0 estimates for each wave based on conversational contacts only. Contacts are restricted to those who were conversational contacts for Waves 1 and up (n = 3,163 in Wave 0, n = 7,363 in Wave 1, n = 7,719 in Wave 2 and n = 11,199 in Wave 3). 95% confidence intervals were derived from the bootstrap. FB: the 2015 Facebook survey.

Supplementary note 7: Sensitivity analysis for baseline R_0 value

Our estimate of R_0 for each survey wave was calculated assuming a distribution for R_0 for COVID-19 in the absence of physical distancing. In the main text, we assume that R_0 prior to physical distancing followed a normal distribution with mean 2.5 and standard deviation of 0.54 based on estimates from literature.[5,26] Since there is a wide range of R_0 estimates in published literature, we repeated the analysis for estimating R_0 under physical distancing with higher and lower estimates of baseline R_0 values. We used the range of estimates of state-level R_0 (in the absence of physical distancing) reported in.[29] We find that for the highest baseline R_0 estimated by Pitzer et al[29] (5.17 for Missouri), the reduction in contacts is not sufficient to reduce R_0 to below 1 in Wave 0; otherwise the results remain qualitatively similar.

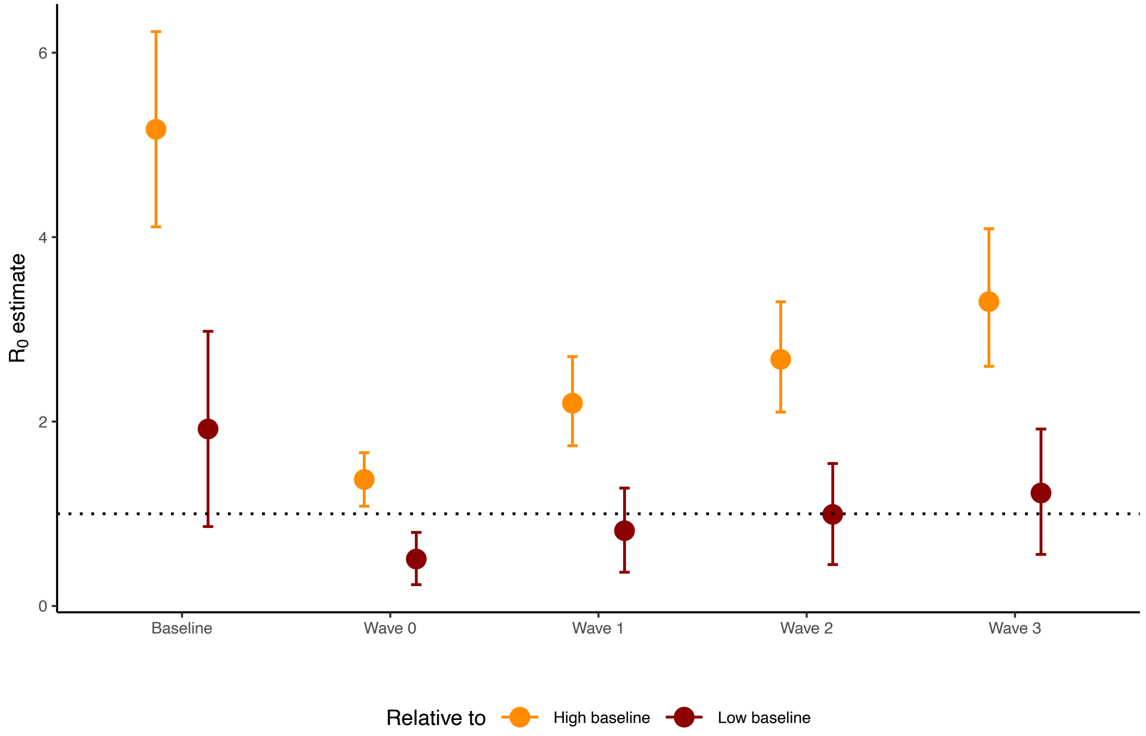

Supplementary figure 6: R_0 estimates from the BICS contact matrices for each wave relative to two baseline R_0 values. The implied R_0 from the BICS contact matrices for each wave is calculated from age-structured contact matrices for all reported contacts (n = 3,163 in Wave 0, n = 7,473 in Wave 1, n = 7,842 in Wave 2 and n = 11,402 in Wave 3), and assuming that baseline contact patterns is equivalent to the 2015 Facebook study. The high baseline R_0 value is drawn from a normal distribution with mean 5.17 and standard deviation of 0.54. The low baseline R_0 value is drawn from a normal distribution with mean 1.92 and standard deviation of 0.54. 95% confidence intervals were derived from the bootstrap.

Supplementary note 8: Comparison of business-as-usual age-structured contact matrices

In the main text, we compare the BICS age-structured contact matrices to a baseline business-as-usual scenario from a 2015 study,[9] based on a probability sample of US Facebook users. As a sensitivity analysis,

we also estimate relative changes in R_0 assuming that baseline contact patterns were equivalent to contact patterns from the UK POLYMOD study,[3] which has been widely used in many settings. Contact patterns in both studies are based on empirical estimates, similar to the BICS study. In the absence of empirical data, however, contact patterns are often derived from model-based estimates that take into account the demographic structure of the population and empricical data from other populations. We compared the contact patterns from Feehan and Cobb[9] and from Mossong et al[3] to model-based estimates of business-as-usual contact patterns in the US from Prem et al[8]. Supplementary figure 7 shows the three contact matrices, and the average number of contacts by age group. We see very similar patterns of assortativeness of contacts by age, although the absolute levels of contact differ slightly across the three studies.

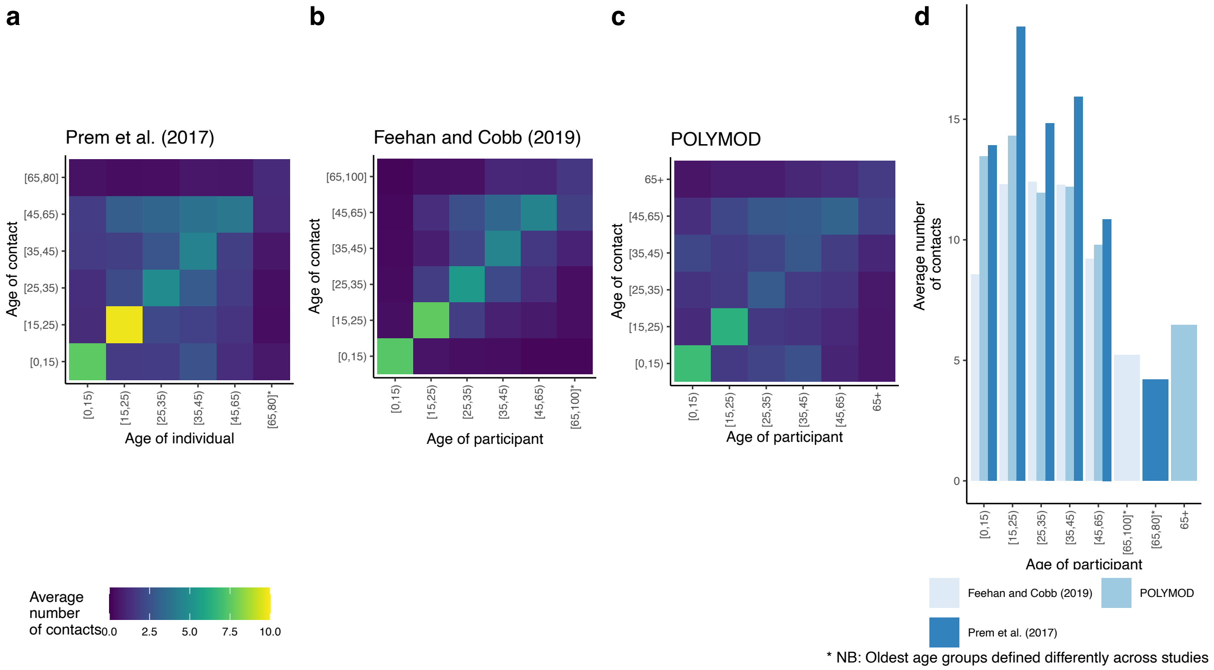

Supplementary figure 7: Comparison of age-structured contact matrices from **a** Prem et al,[8] **b** Feehan and Cobb,[9] and **c** Mossong et al.[3] **d** shows the average number of reported contacts for each age-group in the three studies.

Supplementary references

1. Mervosh, S., Lu, D. & Swales, V. See Which States and Cities Have Told Residents to Stay at Home. *The New York Times* (2020).
2. Davies, N. G. *et al.* Age-dependent effects in the transmission and control of COVID-19 epidemics. *Nature Medicine* 1–7 (2020) doi:10.1038/s41591-020-0962-9.
3. Mossong, J. *et al.* Social Contacts and Mixing Patterns Relevant to the Spread of Infectious Diseases. *PLOS Medicine* **5**, e74 (2008).

4. Wallinga, J., Teunis, P. & Kretzschmar, M. Using Data on Social Contacts to Estimate Age-specific Transmission Parameters for Respiratory-spread Infectious Agents. *American Journal of Epidemiology* **164**, 936–944 (2006).

5. Jarvis, C. I. *et al.* Quantifying the impact of physical distance measures on the transmission of COVID-19 in the UK. *BMC Medicine* **18**, 124 (2020).

6. Zagheni, E. *et al.* Using Time-Use Data to Parameterize Models for the Spread of Close-Contact Infectious Diseases. *American Journal of Epidemiology* **168**, 1082–1090 (2008).

7. Dorélien, A., Ramen, A. & Swanson, I. Analyzing the demographic, spatial, and temporal factors influencing social contact patterns in the U.S. And implications for infectious disease spread. 2020. (2020).

8. Prem, K., Cook, A. R. & Jit, M. Projecting social contact matrices in 152 countries using contact surveys and demographic data. *PLOS Computational Biology* **13**, e1005697 (2017).

9. Feehan, D. M. & Cobb, C. Using an Online Sample to Estimate the Size of an Offline Population. *Demography* **56**, 2377–2392 (2019).

10. Zhang, J. *et al.* Changes in contact patterns shape the dynamics of the COVID-19 outbreak in China. *Science* **368**, 1481–1486 (2020).

11. Latsuzbaia, A., Herold, M., Bertemes, J.-P. & Mossong, J. Evolving social contact patterns during the COVID-19 crisis in Luxembourg. *PLOS ONE* **15**, e0237128 (2020).

12. Fava, E. D. *et al.* The differential impact of physical distancing strategies on social contacts relevant for the spread of COVID-19. *medRxiv* 2020.05.15.20102657 (2020) doi:10.1101/2020.05.15.20102657.

13. Eames, K. T. D., Tilston, N. L., Brooks-Pollock, E. & Edmunds, W. J. Measured Dynamic Social Contact Patterns Explain the Spread of H1N1v Influenza. *PLOS Computational Biology* **8**, e1002425 (2012).

14. Grijalva, C. G. *et al.* A Household-Based Study of Contact Networks Relevant for the Spread of Infectious Diseases in the Highlands of Peru. *PLOS ONE* **10**, e0118457 (2015).

15. Ibuka, Y. *et al.* Social contacts, vaccination decisions and influenza in Japan. *J Epidemiol Community Health* **70**, 162–167 (2016).

16. Eames, K. T. D., Tilston, N. L., White, P. J., Adams, E. & Edmunds, W. J. The impact of illness and the impact of school closure on social contact patterns. *Health technology assessment (Winchester, England)* **14**, 267–312 (2010).

17. Klepac, P., Kissler, S. & Gog, J. Contagion! The BBC Four Pandemic The model behind the documentary. *Epidemics* **24**, 49–59 (2018).

18. Dorélien, A. M. *et al.* Minnesota Social Contacts and Mixing Patterns Survey with Implications for Modelling of Infectious Disease Transmission and Control. *Survey Practice* **Forthcoming**, 13669.

19. Elliott, M. R. & Valliant, R. Inference for Nonprobability Samples. *Statistical Science* **32**, 249–264 (2017).

20. Deville, J.-C. & Särndal, C.-E. Calibration estimators in survey sampling. *Journal of the American statistical Association* **87**, 376–382 (1992).

21. Särndal, C.-E. & Lundström, S. *Estimation in Surveys with Nonresponse.* (John Wiley & Sons, 2005).

22. Ruggles, S. *et al.* IPUMS USA: Version 10.0. (2020) doi:10.18128/D010.V10.0.

23. Sood, G. Geographic information on designated media markets. (2016).

24. Arregui, S., Aleta, A., Sanz, J. & Moreno, Y. Projecting social contact matrices to different demographic structures. *PLOS Computational Biology* **14**, e1006638 (2018).

25. Farrington, C. P., Kanaan, M. N. & Gay, N. J. Estimation of the basic reproduction number for infectious diseases from age-stratified serological survey data. *Journal of the Royal Statistical Society: Series C (Applied Statistics)* **50**, 251–292 (2001).

26. Anderson, R. M., Heesterbeek, H., Klinkenberg, D. & Hollingsworth, T. D. How will country-based mitigation measures influence the course of the COVID-19 epidemic? *The Lancet* **395**, 931–934 (2020).

27. Feehan, D. & Mahmud, A. Replication Data for: Quantifying population contact patterns in the United States during the COVID-19 pandemic. (2020) doi:10.7910/DVN/M74AJ4.

28. Feehan, D. M. & Ayesha S. Mahmud. Dfeehan/bics-paper-release: Live version. (2020) doi:10.5281/zenodo.4323398.

29. Pitzer, V. E. *et al.* The impact of changes in diagnostic testing practices on estimates of COVID-19 transmission in the United States. *medRxiv* (2020) doi:10.1101/2020.04.20.20073338.
