# Supplementary material for: Quantifying population contact patterns in the United States during the COVID-19 pandemic
Source: Nat Commun. 2021 Feb 9;12:893. doi: 10.1038/s41467-021-20990-2 (PMC7873309; doi:10.1038/s41467-021-20990-2)
Supplement: Supplementary file 3 — Reporting Summary [file 41467_2021_20990_MOESM3_ESM.pdf]

nature research

Corresponding author(s): Dennis Feehan

Last updated by author(s): Dec 12, 2020

Reporting Summary

Nature Research wishes to improve the reproducibility of the work that we publish. This form provides structure for consistency and transparency in reporting. For further information on Nature Research policies, see our Editorial Policies and the Editorial Policy Checklist.

Statistics

For all statistical analyses, confirm that the following items are present in the figure legend, table legend, main text, or Methods section.

n/a	Confirmed	
☐	☒	The exact sample size (n) for each experimental group/condition, given as a discrete number and unit of measurement
☐	☒	A statement on whether measurements were taken from distinct samples or whether the same sample was measured repeatedly
☒	☐	The statistical test(s) used AND whether they are one- or two-sided<br>*Only common tests should be described solely by name; describe more complex techniques in the Methods section.*
☐	☒	A description of all covariates tested
☒	☐	A description of any assumptions or corrections, such as tests of normality and adjustment for multiple comparisons
☐	☒	A full description of the statistical parameters including central tendency (e.g. means) or other basic estimates (e.g. regression coefficient) AND variation (e.g. standard deviation) or associated estimates of uncertainty (e.g. confidence intervals)
☒	☐	For null hypothesis testing, the test statistic (e.g. F, t, r) with confidence intervals, effect sizes, degrees of freedom and P value noted<br>*Give P values as exact values whenever suitable.*
☐	☒	For Bayesian analysis, information on the choice of priors and Markov chain Monte Carlo settings
☒	☐	For hierarchical and complex designs, identification of the appropriate level for tests and full reporting of outcomes
☒	☐	Estimates of effect sizes (e.g. Cohen's d, Pearson's r), indicating how they were calculated

Our web collection on statistics for biologists contains articles on many of the points above.

Software and code

Policy information about availability of computer code

Data collection

We created our survey instrument in the Qualtrics survey platform.

Data analysis

Our code is available on GitHub (https://github.com/dfeehan/bics-paper-release) and also in the replication archive on the Harvard Dataverse (https://doi.org/10.7910/DVN/M74AJ4).
We conducted our analyses with the R statistical package and the regression models were fit using stan. The DESCRIPTION file and the Dockerfile in the repository we release can be used to reproduce our software environment by building a Docker image.
We used the versions of the following packages that were available from the CRAN R repository on Dec 11, 2020:
autumn,
bayesplot,
brms,
broom,
broom.mixed,
cowplot,
dplyr,
extrafont,
flexdashboard,
furrr,
future,
ggplot2,
ggpubr,
ggthemes,
glue,

grid,
gt,
gtsummary,
here,
Hmisc,
ipumsr,
janitor,
kableExtra,
knitr,
patchwork,
piggyback,
purrr,
reshape2,
rmarkdown,
rstanarm,
scales,
socialmixr,
survey,
tictoc,
tidybayes,
tidymodels,
tidyverse,
viridis

These can be obtained from the MRAN archive corresponding to December 11, 2020 (https://mran.microsoft.com/) or automatically by using the Docker image we release with our code.
We also used specific versions of the packages autumn and kableExtra:
aaronrudkin/autumn@661698712a44c6a674756e707e5c1cf8751bbb1e,
haozhu233/kableExtra@c3c96bb3c5213781d9db3ef66db5e64d52f1f213
These can be installed in R using, for example,
devtools::install_github("aaronrudkin/autumn@661698712a44c6a674756e707e5c1cf8751bbb1e")

For manuscripts utilizing custom algorithms or software that are central to the research but not yet described in published literature, software must be made available to editors and reviewers. We strongly encourage code deposition in a community repository (e.g. GitHub). See the Nature Research guidelines for submitting code & software for further information.

Data

Policy information about availability of data

All manuscripts must include a data availability statement. This statement should provide the following information, where applicable:
- Accession codes, unique identifiers, or web links for publicly available datasets
- A list of figures that have associated raw data
- A description of any restrictions on data availability

The data we collected and analyzed for this paper are available from the Harvard Dataverse (https://doi.org/10.7910/DVN/M74AJ4).
To replicate our comparisons to Prem et al (2017), you must download this data from https://doi.org/10.1371/journal.pcbi.1005697.s002. The contact matrix for the US (for all locations) is available in a tab in the `MUestimates_all_locations_2.xlsx` file in the downloaded folder.
The POLYMOD data can be downloaded using the R package socialmixr (https://github.com/sbfnk/socialmixr)

Field-specific reporting

Please select the one below that is the best fit for your research. If you are not sure, read the appropriate sections before making your selection.

☐ Life sciences ☒ Behavioural & social sciences ☐ Ecological, evolutionary & environmental sciences

For a reference copy of the document with all sections, see nature.com/documents/nr-reporting-summary-flat.pdf

Behavioural & social sciences study design

All studies must disclose on these points even when the disclosure is negative.

Study description	We collect multiple waves of survey data; this is a quantitative, repeated cross-sectional design
Research sample	In order to estimate rates of interpersonal interaction among people in the US, we interview US adults using an online panel with a quota sample. We have an oversample of respondents from the same online panel in six US cities. Our quota sample design produced respondents whose distribution is proportional to the US national population in terms of age and sex. This is important to ensure that we can study patterns of interpersonal contact by age and sex. We further use calibration weights to improve the representativeness of our sample.
Sampling strategy	We use a quota sample from an online panel.

Data collection	Survey respondents filled out a survey created using Qualtrics. Researchers were not blinded to the study purpose. This was not a randomized experiment whose goal was to test a specific hypothesis, but an observational study whose goal was to document rates of interpersonal interaction.
Timing	Data collection took place in four waves: between March 22 and April 8, 2020 (pilot study, Wave 0); between April 10 and May 4, 2020 (Wave 1); between June 17 and 23, 2020 (Wave 2); and between September 11 and 26 (Wave 3). We surveyed a total of 9,743 respondents in the U.S. (Wave 0 n=1,437, Wave 1 n=2,627, Wave 2 n=2,431, Wave 3 n=3,248).
Data exclusions	No data were excluded from the analyses.
Non-participation	Panel members opted into the study; none refused after opting in.
Randomization	Participants were not allocated to experimental groups.

Reporting for specific materials, systems and methods

We require information from authors about some types of materials, experimental systems and methods used in many studies. Here, indicate whether each material, system or method listed is relevant to your study. If you are not sure if a list item applies to your research, read the appropriate section before selecting a response.

Materials & experimental systems

n/a	Involved in the study
☒	☐ Antibodies
☒	☐ Eukaryotic cell lines
☒	☐ Palaeontology and archaeology
☒	☐ Animals and other organisms
☐	☒ Human research participants
☒	☐ Clinical data
☒	☐ Dual use research of concern

Methods

n/a	Involved in the study
☒	☐ ChIP-seq
☒	☐ Flow cytometry
☒	☐ MRI-based neuroimaging

Human research participants

Policy information about studies involving human research participants

Population characteristics	See above.
Recruitment	Participants were members of an online panel. Members of the panel were offered the opportunity to participate in our study, with targeting for potential participation based on the quota sample criteria, which included age, sex, race/ethnicity, geography. Our sample is somewhat more educated and more urban than the US average. As we describe in the paper, we use weighting to improve the representativeness of our sample.
Ethics oversight	The study was approved by the UC Berkeley IRB (Protocol 2020-03-13128). All survey participants provided informed consent before responding to the survey.

Note that full information on the approval of the study protocol must also be provided in the manuscript.
